# Full Mouth Rehabilitation of Two Siblings with Dentinogenesis Imperfecta Type II Using Different Treatment Modalities

**DOI:** 10.3390/ijerph17197029

**Published:** 2020-09-25

**Authors:** Murad Alrashdi, Jason Schoener, Claudia Isabel Contreras, Shuo Chen

**Affiliations:** 1 Department of Orthodontic and Pediatric Dentistry, College of Dentistry, Qassim University, Buraydah 51452, Saudi Arabia; 2 Royal Air Force Lakenheath, Brandon IP27 9PS, UK; jason.d.schoener@gmail.com; 3 Department of Developmental Dentistry, School of Dentistry, University of Texas Health Science Center at San Antonio, San Antonio, TX 78229, USA; contrerasc2@uthscsa.edu (C.I.C.); chens0@uthscsa.edu (S.C.)

**Keywords:** dentinogenesis imperfecta, dental anomaly, DGI type II

## Abstract

Background: Dentinogenesis imperfecta (DGI) is a complex anomaly, not only by its structure but by treatment approach. The treatment protocol depends on the severity, behavior, and the age of the patient. Case Description: This paper presents two siblings’ cases of DGI type II (DGI-II) with different treatment based on the patient’s clinical severity, behavior, and age (mixed versus primary dentition). The first case involves a patient in the primary dentition with severe attrition leading to a reduction in the vertical dimension of occlusion (VDO) treated by the fabrication of complete overlay dentures. The second case involves a patient in the early mixed dentition treated with restorations and extractions. Conclusion: Full mouth rehabilitation in the two patients dramatically improves function, aesthetics, and proved to be a significant psychological boost to the patient’s well-being. Practical Implications: Early diagnosis and a multidisciplinary approach for patients with DGI to preserve the remaining teeth and rehabilitation for their function and aesthetics are essential for obtaining a favorable prognosis.

## 1. Introduction

Dentinogenesis imperfecta (DGI) is a genetic disorder in tooth development that affects both the primary and permanent dentition. DGI patients exhibit teeth with blue–gray or yellow–brown colors that are prone to wear, fracture, and loss [1]. As DGI is an autosomal dominant inherited condition, there is a 50% chance the child will suffer from the disease when one of the parents has this genetic disorder. DGI is one of the most common dentin genetic disorders in humans, present in one out of every 6000 to 8000 births [2,3]. Shields et al. classified DGI into three types; DGI type I (DGI-I), which is associated with osteogenesis imperfecta (OI) due to collagen gene mutations, DGI type II (DGI-II), which has similar clinical, radiographic, and histological features but without OI, and DGI type III (DGI-III), which is rare and only found in the triracial Brandywine population of Maryland [4]. It is known that DGI-II and DGI-III are associated with a dentin sialophospho protein (DSPP) gene mutation, which is highly expressed in odontoblasts and located on chromosome 4q21.3 [5]. Differential diagnoses include dentin dysplasia, hypocalcified forms of amelogenesis imperfecta, congenital erythropoietic porphyria, conditions leading to early tooth loss (Kostmann’s disease, cyclic neutropenia, Chediak–Hegashi syndrome, histiocytosis X, Papillon–Lefevre syndrome), permanent teeth discoloration due to tetracyclines, Vitamin D-dependent and vitamin D-resistant rickets [1].

DSPP is proteolytically cleaved into dentin sialoprotein (DSP) and phosphorphoryn (PP). The study by Zhang et al. found that the interaction between DSP and PP proteins may be necessary for dentinogenesis [6], which is disrupted in DGI, while Qui et al. demonstrated that proteolytic cleavage of DSPP is a necessary activation step in the formation of dentin [7]. Furthermore, in DGI patients without DSPP cleavage, dentin pulp cells differentiate into chondrocyte-like cells, which negatively impact pulpal wound healing (e.g., secondary and tertiary dentin) and tissue regeneration [8].

The dentino-enamel junction connects through scalloping morphological peculiarity that leads to the mechanical interlocking between the enamel and dentinal tissues [9,10]. Due to changes in the dentin structure in DGI patients, the affected dentin lacks scalloping, leading to the fracture of enamel, quickly forming the defective dentin [11].

These case studies aim to present the objectives, treatment, and problems encountered in the treatment of DGI-II children from the same family, using two different treatment approaches, one in primary dentition and the other in the early mixed dentition.

## 2. Case One

A 5-year-old girl presented with her mother to the University of Texas Health San Antonio School of Dentistry Graduate Pediatric Dental Clinic. The child had multiple prior visits to the clinic for emergency treatment of abscessed teeth. This time, the mother’s chief complaint was, “my daughter is going to school next year, and I am worried about her being harassed due to the color of her teeth and the way her face looks”.

**Figure 1:**
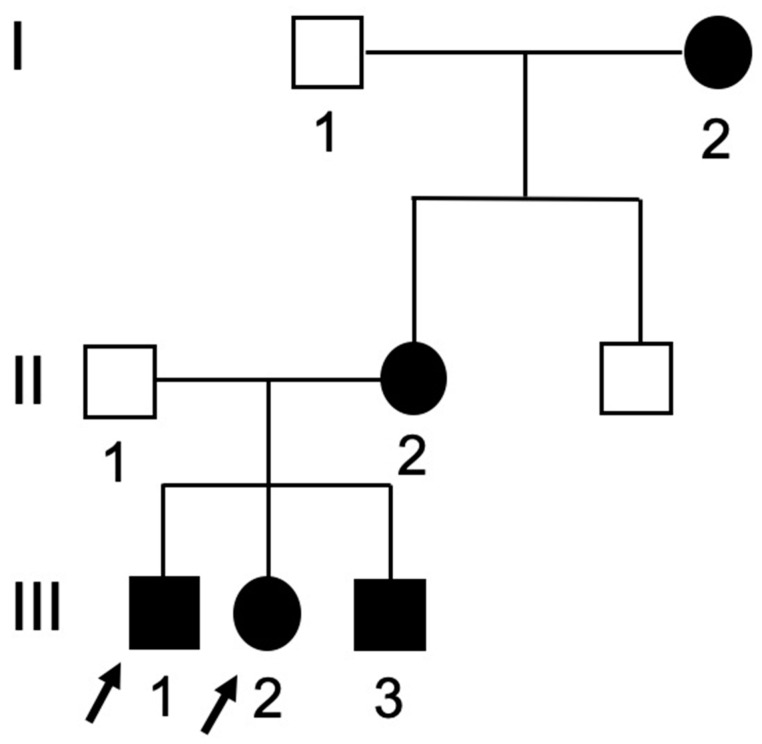
Pedigree of the dentinogenesis imperfecta type-II (DGI-II) family with arrows indicating the probands of the family. Darkened = affected; clear = unaffected, squares = males; circles = females. I = 1st generation; II = 2nd generation; III = 3rd generation.

The girl was born after a full-term pregnancy, and all milestones of development were normal. There was no history of unusual bone brittleness, drug use in the present or past, or any other systemic illness. The patient had fair oral hygiene with a limited frequency of consumption of sugary food and drinks. Family history revealed that the mother was diagnosed with DGI-II and wore an implant overdenture. Moreover, two of her siblings would later receive a diagnosis of DGI-II. The pedigree of the DGI-II family is shown in Figure 1. The third affected sibling was almost two years old and not included in this case report.

**Figure 2:**
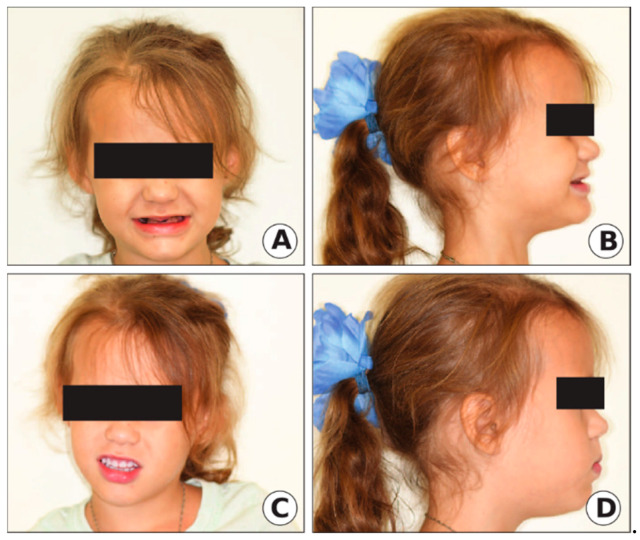
Clinical extraoral photographs of Case 1. (**A**,**B**) Before treatment. (**C**,**D**) After (overlay denture) treatment.

**Figure 3:**
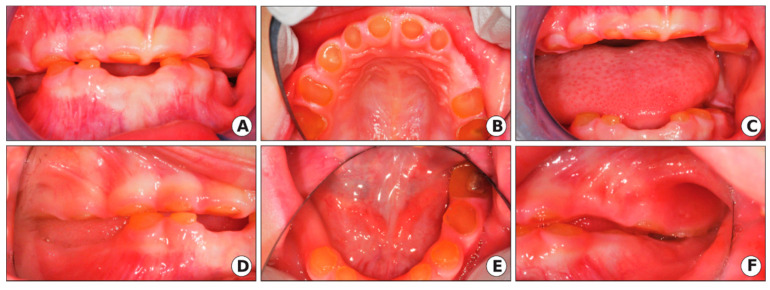
Clinical oral manifestation of DGI-II in the proband of the 5-year-old girl of the DGI-II family. The intraoral photographs showed severe attrition of the primary dentition to the gingiva level with color deviation from normal to yellow–brown with a translucent appearance. (**A**) Intraoral photo of the anterior view (mouth closed). (**B**) Intraoral photo of the maxillary arch. (**C**) Intraoral photo of the anterior view (mouth open). (**D**) Intraoral photo (right side), (**E**) Intraoral photo of the mandibular arch. (**F**) Intraoral photo (left side).

The extraoral exam revealed a loss of vertical dimension of occlusion (VDO) with a typical appearance of an edentulous person, decreased lower facial height, prognathic facial profile, and loss of upper and lower lip support (Figure 2a,b). The intraoral exam showed severe attrition/wear of the primary dentition to the level of gingiva with color deviation from normal to yellow–brown with a translucent appearance, as seen in Figure 3.

**Figure 4:**
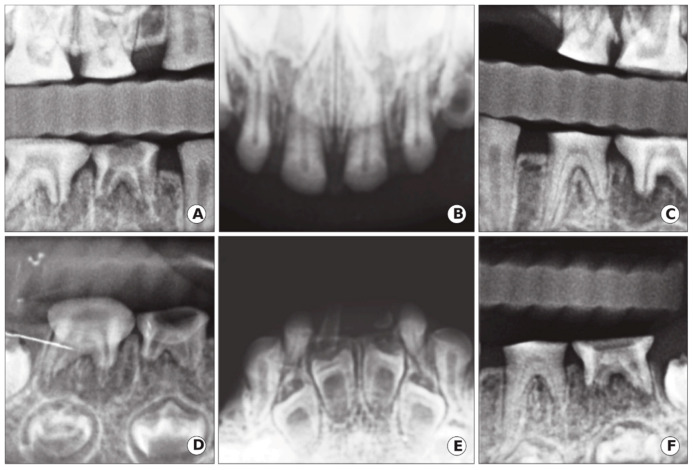
Case 1: Radiographic oral manifestation of DGI-II in the proband of the 5-year-old girl of the DGI-II family. (**A**,**C**) Bitewings radiographs showed that dentine was thin with severe occlusal attrition. (**B**,**E**) Upper and lower occlusal radiographs showed incisal attrition with narrowing of the pulp, in addition to enlarge pulp chambers and thin enamel in the developing lower anterior permanent teeth. (**D**,**F**) Periapical radiographs of the lower right and left molars demonstrated occlusal attrition and abscessed second right and left molars.

Radiographic examinations revealed a primary dentition present with severe loss of the enamel associated with severe occlusal attrition. Primary upper and lower left second molars, and primary right first and second molars had pulpal involvement with a periapical radiolucency. The presence of developing permanent buds was also evident with thin enamel, large pulp chambers, and mild cervical constriction of the crowns. The child’s dental age, determined radiographically, was consistent with her chronological age (Figure 4).

After correlating the clinical and radiographic findings, along with positive family history, the diagnosis of DGI-II was suspected. Since the crown structure of all teeth had severe attrition and the patient lost VDO, in addition to the mother chief complaint, it was decided to extract the non-restorable teeth that had periapical radiolucencies (upper and lower left second primary molars and lower right first and second primary molars) and fabricate an overlay denture, while increasing the VDO for this patient to restore the smile as shown in Figure 2c,d. To confirm the diagnosis of DGI-II, the extracted teeth were sent to a laboratory for histological analysis, and blood samples were obtained from the mother of the child for DNA sequencing.

After two months of healing at the extraction sites, a diagnostic impression was obtained with nitrous oxide (due to mild dental anxiety) to fabricate a custom-made tray. Genie ™ putty vinyl polysiloxane (VPS) impression material, berry-flavored (Sultan Healthcare, Hackensack, NJ, USA), was used because it is a rapid set, which allowed the impression to set faster. Thermoplastic (suck down) base plates with wax occlusal rims were made, and the VDO was established by increasing it around 6 mm. The cast was mounted on a fixator articulator, and the over denture was fabricated over all the remaining primary teeth as an abutment. Primary acrylic teeth were selected using the JBC Company, Bambino’s teeth, SHADE 5/59 (JBC and Company, Fredericksburg, TX, USA). Teeth were arranged in wax with normal physiological spacing and rounded free gingival margins. The upper denture fitted well at the initial try-in, but the lower denture was re-impressed for better stability. After the second satisfactory try-in, the denture was processed for final delivery.

Histological analysis/DNA sequencing was performed, one of the extracted teeth was used to isolate primary mesenchymal cells, and the other was fixed for histology study. Genomic DNAs were isolated from these cells and performed polymerase chain reactions (PCR) to amplify the human DSPP gene, and then the PCR products were subcloned into the PCR-II vector. The PCR machine used was T100TM, thermal cycler, Bio-Rad Laboratories, Inc. 2000 Alfred Noble Drive, Hercules, CA, USA. The inserted human DSPP gene was sequenced by the Sanger sequencing machine (GENEWIZ Global Headquarters, South Plainfield, NJ, USA) using specific human DSPP gene primers. A partial DSPP gene was identified compared to databases from the National Institutes of Health (NIH) blast search, and polymorphisms DSPP gene in the patient was found (data not shown).

## 3. Case Two

A 7-year-old male presented to the same clinic with his mother and younger sibling noted in case one. The patient had a severe autism spectrum disorder (ASD), DGI-II, and sickle cell trait (SCT). The child was non-verbal and unable to report tooth pain. The patient mixed dentition, with a past history of primary teeth extracted under oral conscious sedation (OCS). The patient had poor oral hygiene with large amounts of plaque and calculus build up on anterior teeth.

**Figure 5:**
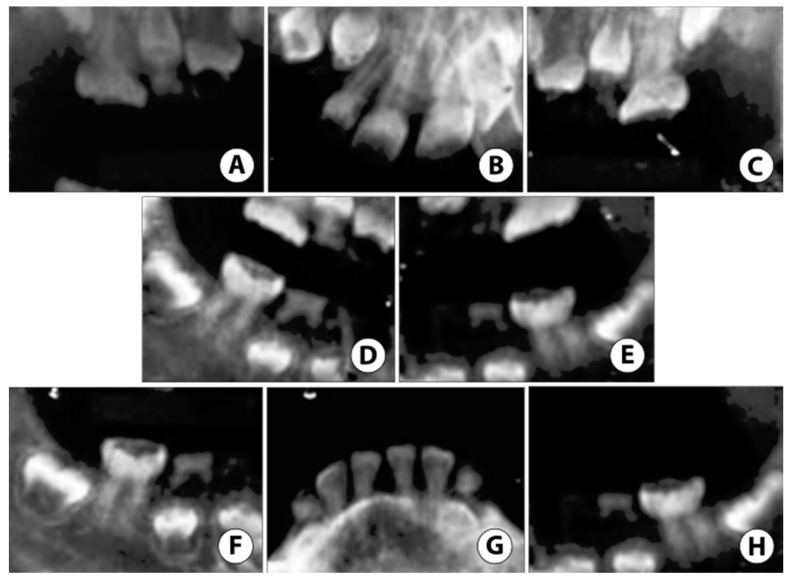
Case 2: Non-digital radiographs taken in the hospital under General Anesthesia (GA) reveal (**A**–**C**) severe enamel loss with reduced pulp space in both primary and permanent teeth. (**D**,**E**) Root tip fragments of upper primary canines noted. (**F**,**H**) Extensive atypical occlusal caries noted on lower permanent first molars. (**G**) Reduced pulp space in permanent mandibular incisors with near exfoliation of lower primary canines.

**Figure 6:**
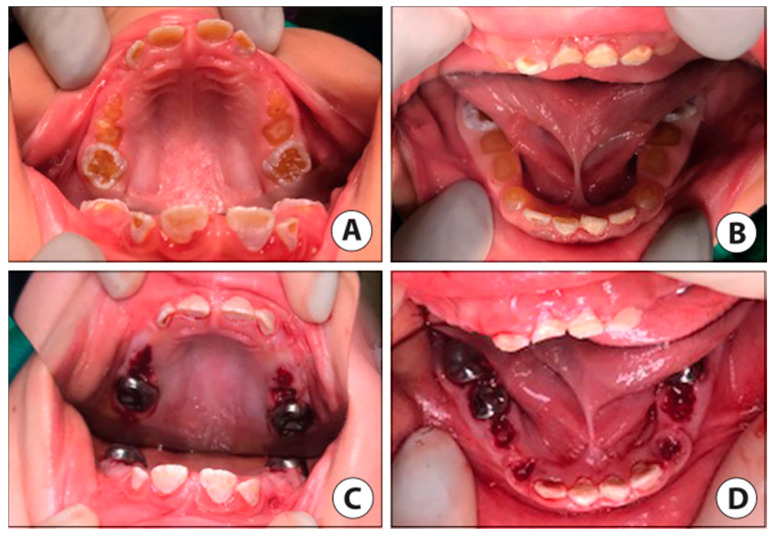
Case 2 clinic photos. (**A**) Pre-op intraoral photo of the maxillary arch. (**B**) Pre-op intraoral photo of the mandibular arch (mandibular first molars partially visible). (**C**) Post-op intraoral photo maxillary arch reveals stainless steel crowns (SSC) restorations permanent first molars, facial resin-modified glass ionomer (RMGI) restorations, lingual sealants on permanent incisors, and blood clots at extraction sites of all remaining primary teeth. (**D**) Post-op intraoral photo mandibular arch reveals SSCs placed on permanent first molars (lower left permanent first molar not visible) and second primary molars, with the extraction of primary first molars and canines. RMGI placed on facial surfaces of anterior incisors.

Due to prior ineffective OCS and severe ASD, treatment under General Anesthesia (GA) was accepted as the best method to restore the patient’s oral health. Under GA, a full set of radiographs were taken (Figure 5), along with intraoral photographs (Figure 6), which revealed an irregular/extensive caries pattern extending to the cusp on all permanent first molars due to attrition and poor enamel to dentin strength associated with DGI-II. The patient had amber-hue colored anterior teeth with most enamel missing and attrition down to the gingival margin on all primary first and second molars, and caries were present on the facial surface of all upper and lower anterior incisors as seen in Figure 6.

Primary teeth were extracted due to mobility, as seen in the primary canines and severe, non-restorable attrition in primary molars, except the mandibular second primary molars, which were considered stable enough for stainless steel crowns (SSC) placement. SSCs are thin metal prefabricated caps used to protect multiple tooth surfaces and can be placed in one appointment without the use of a dental laboratory. All permanent first molars were restored with SSC (3M ESPE, St. Paul, MN, USA) due to an atypical caries pattern and poor enamel quality. Anterior permanent incisors were restored with facial surface Fuji II LC, a resin-modified glass ionomer (RMGI) (GC America, Alsip, IL, USA) due to better quality enamel margins and the lingual surfaces of the incisors had UltraSeal XT ^®^ hydro ^TM^ sealant (Ultradent Products, South Jordan, UT, USA) placed due to previous food entrapment in the deep lingual fossa of the incisors. At the follow up appointment, the mother said the patient was eating well and following improved oral hygiene due to motivation from the mother and siblings when brushing before bedtime.

## 4. Discussion

One of the most significant challenges in the treatment of the DGI condition in children, besides behavioral management, is to provide adequate treatment to achieve functional and aesthetic restorations. The rational use of advanced behavior management at an early age enables practitioners to safely deliver quality restorative treatment in young children with special health care needs [12]. Tell–show–do, distraction, nitrous oxide, oral conscious sedation, and positive reinforcement were utilized for managing the patient in case one, while the child in case two was treated in the hospital under GA due to severe ASD and high dental anxiety.

In primary teeth affected with DGI-II, the enamel is thin and the dentin exposed early, leading to rapid and severe attrition [13]. Therefore, it is necessary to establish an early diagnosis, along with proper management. In this case report, the two children were presented with two different treatment plans due to their behavior, severity, and age (primary versus mixed dentition), while also addressing the chief complaint of the parent. When the disease is more severe, and teeth are worn down to the gingival level, the biochemical properties of enamel and dentin are compromised, which affects adhesion strength, leaving limited restorative treatment options. For example, in the first case, the child presented to the clinic at a more severe stage of the condition, where all teeth had severe attrition, and some of them were abscessed and non-restorable. In that case, the patient’s best long-term treatment option was a complete overlay denture to address the child’s condition and the mother’s chief complaint. An overlay denture is a partial or complete removable denture fabricated over retained teeth or roots of the teeth that are not prepared with any coping to interface with the denture [14]. If this patient had been seen at an earlier stage, intervention to perform full mouth dental rehabilitation of the primary teeth by restoring the teeth with SSC and esthetic prefabricated crowns could have been performed. Therefore, early diagnosis and education of parents with DGI is important so that they are aware of the different issues that will occur as their children transition into permanent dentition.

The second patient presented in the early mixed dentition with the recent eruption of permanent teeth allowing us to restore the posterior teeth with SSCs and the anterior teeth with esthetic restorations. Both of the patients were placed on a regular recall for their oral examination (every 6 months), dental prophylaxis, oral hygiene instruction, and, in the first case, denture adjustment, if necessary, as the child’s growth may necessitate relining or remaking the denture [14]. Ideally, follow up care should be maintained until an implant can be placed when growth is completed [15]. Regarding the fabrication of the overlay denture, another option was to incorporate an expansion screw, and if there were a need, to begin to expand to allow growth in the transverse dimension. This limits having to remake the appliance in its totality.

## 5. Conclusions

Early diagnosis and treatment of DGI are essential for obtaining a favorable prognosis. A multidisciplinary approach to preserve remaining teeth and rehabilitation of the function and aesthetic is of utmost importance. Prosthodontic rehabilitation of a child with severe DGI in the late stage of the primary dentition significantly improves function, aesthetics and proves to be a significant psychological boost to the patient’s well-being. While there is no known link to ASD and SCT with DGI, we see the importance of future research in this area.
